# Implementing advance care planning in Swedish healthcare settings – a qualitative study of professionals’ experiences

**DOI:** 10.1080/02813432.2022.2155456

**Published:** 2022-12-15

**Authors:** Simon Beck, Lina Lundblad, Camilla Göras, Malin Eneslätt

**Affiliations:** a School of Health and Welfare, Dalarna University, Falun, Sweden; b Department of Health, Education, and Technology, Luleå University of Technology, Luleå, Sweden; c Department of Learning, Informatics, Management, and Ethics, Karolinska Institutet, Stockholm, Sweden

**Keywords:** Advance care planning, nursing homes, health plan implementation, patient-centred care, long-term care, implementation science

## Abstract

**Background:**

Advance care planning (ACP) is a process involving conversations about values and preferences regarding future care at the end-of-life. ACP has led to positive outcomes, both in relation to quality of life and with increased use of palliative care, less life-sustaining treatment and fewer hospital admissions. Sweden has yet to embrace the practice systematically, but scattered initiatives exist.

**Aim:**

To study implementation of a routine for ACP in NH settings in Sweden by exploring healthcare professionals’ experiences of engaging in ACP following this implementation.

**Methods:**

The study followed a qualitative inductive design with convenience and snowball sampling. Semi-structured group and individual interviews with registered healthcare professionals were analysed using qualitative content analysis.

**Findings:**

Organisational support for sustainable ACP implementation was found to be essential. This included sufficient training, facilitation, collaboration and uniform work routines across providers and professionals. Engaging in ACP conversations following the implemented routine was found to be a process of preparing, being, talking, deciding and sharing.

**Conclusions:**

Successful implementation of ACP in NHs requires a carefully planned implementation strategy. ACP in NHs tend to be medically focused at the expense of residents’ psychosocial care-planning needs. Widespread uptake of ACP in Sweden could be useful in the national effort to adopt more person-centred care in Swedish healthcare.

**KEY POINTS**
 While advance care planning has been implemented in many other countries, Sweden lacks a national strategy on advance care planning and Swedish healthcare settings have yet to systematically implement this practice.
  • This study is the first to report on professionals’ experiences of engaging in sustainable advance care planning, following top-down implementation of the practice in one Swedish region.
  • Successful implementation of advance care planning in nursing homes requires a system-level approach, and shortcomings of the implementation process are highlighted.

## Background

Advance care planning (ACP) has been defined as a process of reflection and conversations, which may involve patients, family, and healthcare staff, about values and preferences regarding future care at the end-of-life. ACP can include medical decision making, as well as concerns of a psychological, social and spiritual nature [1]. Scientific discussions on definitions and conceptualisations of ACP, as well as its value, are ongoing [2–4].

ACP can increase delivery of care congruent with individual preferences, and has been seen to reduce undesired hospital admissions [5–8], life-support treatment [8], as well as increase quality of life, healthcare satisfaction, and use of palliative care [5,6,8]. Despite the sensitive nature of ACP conversations, studies show no evidence of increased stress, anxiety, or depression, either for patients or their family [5,6,9].

Many countries have implemented ACP [7] and a number have established legislation around ACP-informed healthcare decisions [10]; such legislation is lacking in Sweden. While scattered ACP-related initiatives have been studied in the general public as well as care settings [11–14], it has been argued that systematised use of ACP in Swedish healthcare systems remains scarce [15]. A retrospective study [16] from another Swedish region on the prevalence of advance care plans in deceased nursing home (NH) residents, however, reported surprisingly high numbers (77–97%). This discrepancy can be explained by the inclusion of palliative care plans formulated within the last month before death, without which figures would have been significantly lower. As mentioned above, discussions on different conceptualisations of ACP are ongoing, and it may be argued that late-stage palliative care plans do not constitute meaningful *advance* care planning [17].

Despite the lack of a nationwide approach to ACP in Sweden, an initiative to implement ACP as a routine procedure in NHs in a northern Swedish region was launched in 2020. In Sweden, over a third of the population die in NHs [18], making them a pertinent setting for ACP; particularly as the ACP process with repeated conversations requires regular contact as well as established relationships between healthcare staff and patients [19]. To guide ACP initiation in the Swedish ACP-naive context, more knowledge is required on issues surrounding implementation of ACP in Swedish healthcare contexts. The aim is thus to study implementation of a routine for ACP in NH settings in Sweden by exploring healthcare professionals’ experiences of engaging in ACP following this implementation.

## Methods

This study followed a qualitative inductive design using group and individual interviews with registered healthcare professionals.

### Setting and sample

In Sweden, municipalities are responsible for providing NHs and home care services for older persons [20]. Municipalities have their own nursing and auxiliary personnel but do not employ physicians, which are provided by the regional healthcare authority [21].

The aforementioned project implementing ACP in NHs in a region in northern Sweden provided the setting for this study. The authors, of which none lived or worked in the target region, carried out the study on the initiative of the project organiser, a general practitioner working there. Mostly unassisted, the project organiser had drafted a policy memorandum [22] on ACP and personally disseminated it region-wide prior to initiation of the current study. The project did not follow an explicit implementation strategy, nor was it a focus of the policy memorandum to guide ACP implementation in the workplace. Rather, the memorandum was directed towards healthcare professionals with instruction on how to carry out ACP, detailing the target group, appropriate timing for ACP conversations, interview technique, decision making and documentation. It recommended an introductory meeting upon residents’ arrival to the NH to map their health status and care wishes; followed by a more thorough and medically oriented ACP conversation two-four weeks later, led by a nurse and/or physician, together with residents and their chosen family. An advance care plan covering both care and medical aspects including decisions to limit treatment (DLTs), symptom alleviation and patients’ own wishes should then be documented, and revisited yearly in connection with a medication reconciliation, or as needed due to changes in health status or on request of residents [22].

Potential participants for the current study were recruited through convenience and snowball sampling, as the target population was known to be small due to the short time frame from project initiation. The inclusion criterion was registered healthcare professionals working with ACP in NHs in the target region. However, participants’ knowledge of ACP was not assessed before enrolment. Recruitment was based on an exhaustive list of all those known by the project organiser to either having begun or were set to begin working with ACP in the region, and comprised contact details of 33 managers, physicians, general and specialist nurses.

These 33 people, together with 20 more who came to our attention via snowball sampling, were contacted by email with information about the study and asked about interest in participation. Fifteen people agreed to be interviewed but four of these later chose not to take part, resulting in eleven participants. Of the eleven, one gave responses in their interview suggesting a less than rudimentary understanding of ACP, leading to their data being excluded. This left interview data from ten people that were included for data analysis (Table 1). Ten NHs are represented in the data out of a total of 91 NHs in the region. The nurse participants were, when on call at weekends and evenings, responsible for more NHs in their area than their primary workplace, and therefore had some insight into the working practices even in other NHs.

**Table 1.** Characteristics of participants (*N* = 10).

Characteristic	
Median age (range)	41 (28–61)
Sex	*n*
Female	6
Male	4
Profession	
Registered nurse	4
Physician	3
NH manager	2
Community health nurse	1
Median years of stated professional experience (range)	10 (4–35)

### Data collection

Our initial intention was to collect data via group interviews, as they are suitable when studying experiences, values and thoughts since group dynamic can support participants to share [23]. However, organising group interviews with professionals turned out to be a challenge and individual as well as group interviews were performed (Table 2).

**Table 2.** Overview of interview forms, participants, and duration.

Interview form	Number of participants	Professions of participants	Duration (min)
Group	3	NH managers (2), registered nurse (1)	52
Group	2	Physician (1), specialist nurse (1)	51
Group	2	Registered nurse (2)	44
Group	2	Registered nurse (2)	41
Individual	1	Physician	30
Individual	1	Physician	23

Prior to being interviewed, participants gave their written informed consent after receiving verbal and written information. Interviews followed a guide composed of four key areas (Supplementary File 1), focusing on: participants’ understandings of ACP and how it should be implemented; their experiences of ACP; and factors that can facilitate or hinder ACP and its implementation. Follow-up questions were used when needed to help clarify or elucidate more meaning from participants. The interview guide was pilot tested with one of the authors’ colleagues, a district nurse who had not personally worked with ACP but had experience of another conversation-based modality and answered based on those experiences. Following pilot interviewing, the guide was judged to be coherent and comprehensible.

Interviews were held during September and October 2021. Due to pandemic restrictions, as well as the geographical distance between participants from different workplaces, interviews were held digitally via a video conferencing platform. Authors LL and SB assumed the roles of moderator and assistant respectively. The moderator led the interviews while the assistant took notes, entering actively into the interview only to ask for elaboration or pose follow-up questions. The conversations were allowed to flow freely but where a participant had not been active in the discussion of a specific question, that question was posed more directly to them. The interviews were audio-recorded and later transcribed verbatim by one interviewer and checked by the other.

### Data analysis

Analysis was carried out manually in a text document by LL and SB, in frequent discussions with CG. Interview data were analysed manifestly using content analysis [24]. The transcripts were read several times by both authors to obtain a sense of the whole. Meaning units were extracted, condensed, and given a code that described their core meaning. Codes were based on similarities and differences and sorted into subcategories which were step-wise aggregated into categories (Table 3). To maintain consistency, there was a movement back and forth between the transcriptions, codes, sub-categories, and categories.

**Table 3.** Overview of categories and subcategories.

Category	Acknowledging organisational support as essential in sustainable ACP implementation		
Subcategories	Knowledge: lack of formal education and in-service training	Conformity: wishes for uniform practices across providers and professionals	Sustainability: challenges in implementing and maintaining ACP routines

Category	Engaging in ACP conversations following an implemented routine				
Subcategories	Preparing: finding the right time and including the right people	Being: using professional competence to bridge difficulties	Talking: a balancing act when juggling sensitive topics and various expectations	Deciding: guiding decision-making	Sharing: documenting preferences and disseminating information

### Ethical considerations

Ethical approval for this study was sought and obtained by the Research Ethics Committee at Dalarna University, Sweden (ID: 7.1.1-2021/292).

### Findings

Analysis resulted in two main categories with three and five subcategories respectively, see Table 3. In presenting our results, we name all participants ‘professionals’ irrespective of profession, except for when deemed important for readers’ understanding.

### Acknowledging organisational support as essential in sustainable ACP implementation

This category concerns organisational level issues in the implementation process, highlighting the lack of ACP training, wishes for uniform practices across providers and professionals, and challenges in implementing and maintaining ACP routines.

### Knowledge: lack of formal education and in-service training

None of the professionals had been taught about ACP during their professional education. Some workplaces had received limited in-service training, while others were directed to the written policy memorandum and left to implement routines without guidance:

We’ve not had any formal training. It’s more that you had to read the document if you were interested, time permitting. So no, not that much training in ACP, I wouldn’t say. More learning by doing, really. (Participant #7, physician)

While the policy memorandum was considered by participants to be well-written with clear instructions, the routines described therein had not been implemented fully in all workplaces. More in-service training was called for, and it was suggested that physicians and nurses be trained together to foster collaboration. Specific training for physicians in writing care plans was also considered important. On-going support, in group reflections or individual mentoring, was deemed necessary if ACP was to be maintained with good standards. Creating conditions for such support structures was considered a managerial responsibility:

But there needs to be a proper forum to discuss these things together. I mean, ethical issues, that kind of thing… And, that has to come from above, because most of the time you’ve got enough on your plate and don’t really have the possibility to… create the space for it. It needs co-ordination by managers to make it easier. (Participant #1, manager and nurse)

### Conformity: wishes for uniform practices providers and professionals

Professionals had experienced large variations in how ACP was approached and carried out, between regionally operated health centres and municipality run NHs, between different NHs in the same municipality, and individually between different professionals. For municipalities and the entire region to work with ACP in the same way, clear leadership was called for. Suggestions included having a facilitator consistently providing support:

So actually, if you were to get the whole of the municipality on board with this, there’d be one person working on implementation, giving support, following how it goes. Because it’s… difficult otherwise. […] There’s no one really taking a hold of it. (Participant #1, NH manager and nurse)

Nurses experienced varying levels of commitment towards ACP from different health centres, making arrangements for physician home visits for ACP conversations a task of varying difficulty. It was considered challenging to implement ACP routines when nurses in NHs were positive towards ACP but partnered health centres were not equally engaged. Some professionals identified risks in nurses taking on duties that were ultimately physicians’ responsibilities. One NH manager recognised their own role in achieving better conformity between NHs and their partnered health centre:

Nursing home managers also need to be on board with this because they’re the ones that negotiate these, you know, collaboration agreements with health centres. They have to pursue the matter. (Participant #1, NH manager and nurse)

While acknowledging the regional policy memorandum on ACP, a shorter document with more concrete instructions and clear demarcations of responsibility was called for to support uniform working practices in the region and its municipalities.

Different NHs, even in the same municipality, were said to have established different routines for how up-to-date information, in particular DLTs, were disseminated to nursing auxiliaries. This was reported to be problematic for nurses on call, who in acute situations need to access DLTs quickly and easily, while relying on auxiliaries for accurate information. It was expressed that there may not always be time for the nurse to search in the documentation system for patients’ care plans:

It’s a major issue here in this municipality […] we want a concrete way how we relay the information, at least [do not resuscitate] decisions, to the care staff. I don’t have to know straight away if [residents] can be sent to the hospital or not. You can wait a few minutes for that and have time to look it up. But you can’t wait a few minutes before starting [cardiopulmonary resuscitation] or not. …so we all do it differently [here]. (Participant #6, nurse)

### Sustainability: challenges in implementing and maintaining ACP routines

ACP routines were introduced in some NHs hastily in early 2020, coinciding with the impending covid-19 pandemic. Hasty implementation, together with lack of in-service training, resulted in worry among some nurses who drew their own conclusions about what ACP would entail:

My very first thought when we were told that ACP was to be brought in here was: ‘oh, how horrible! Is it up to us now to decide whether someone, an individual, should be given help or not, be given [cardiopulmonary resuscitation].?’ Yeah, I had a lot of thoughts rushing around my head then. (Participant #4, nurse)

There was an understanding that ACP had to be actively prioritised to be maintained. While ACP routines had been successfully implemented and maintained in some workplaces, others had seen routines ebb out due to lack of time and resources. Time constraints were seen to be an issue for physicians, having only six minutes per resident per week in which to address all medical questions. Giving more focus to ACP was seen to negatively affect other medical needs. More time allocation was wished for. Another solution to physicians’ time constraints was for nurses to take a greater role in the ACP process or to postpone ACP conversations. It was not agreed by all however that time was an issue. One physician saw it as more a matter of planning:

Everyone who moves into a nursing home has a doctor’s home visit at some point in the first months. And now we put more emphasis on those visits, ask the relatives to come, and make up a care plan during them. And… there must be time available for that. Everywhere, I’d imagine. (Participant #9, physician)

Some nurses reported challenges in having to liaise with multiple physicians, due to being partnered with many health centres. This was challenging on a practical level in communicating with many physicians, but also in different physicians having varying approaches to carrying out ACP.

### Engaging in ACP conversations following an implemented routine

Engaging in ACP conversations was found to be a process of preparing, being, talking, deciding, and sharing.

### Preparing: finding the right time and including the right people

Professionals described how they made preparations for timely ACP conversations, often early after residents move into the NH. This was seen to be important as NH residents’ health can deteriorate quickly, and decision-making was considered easier in a calm and stable phase than in an emergency. It was preferred, however, that time be given for residents to first get accustomed to their new surroundings and for relatives to adapt. This period of adjustment was seen by professionals as valuable for nurses to get to know residents and assess their health status. A suggested appropriate time for ACP conversations was during physicians’ first home visit, usually within the first month of residents moving in.

Relatives’ presence during ACP conversations was valued and recommended, especially with residents suffering from dementia. In-person attendance was preferred, but telephone participation was described as better than non-attendance. Professionals had experienced that sometimes residents and relatives wanted to discuss ACP questions among themselves first and then later discuss it with healthcare personnel, leading to more straightforward conversations. Relatives’ sometimes unrealistic expectations of elderly care was seen by professionals to inhibit ACP conversations from taking place early on in residents’ stay. This was reported to be more common when relatives lived far away and were not up-to-date on how frail or sick their family member had become. Established nurse-relative relationships and discussions held in advance with relatives around expectations of care were seen to be key in mitigating this.

Professionals expressed that ACP conversations could concern medical and nursing issues and stressed the importance of having both those professions present. Presence of nurses who had an established relationship with residents prior to ACP conversations was valued greater by physicians:

It’s so much better when it’s someone who knows the patients. If it’s a temporary nurse, a fill-in, then there’s like no… there’s not much point that they’re there. But if it’s their main responsibility, and they’ve followed the patient, then it’s much more valuable. (Participant #9, physician)

### Being: Using professional competence to bridge difficulties

Professionals claimed that ACP conversations require engagement, sensitivity, and empathy from healthcare personnel. It was expressed that ACP conversations could elicit different kinds of reactions, and that having to meet and deal with those reactions could be psychologically demanding.

What I find difficult with these conversations personally, sometimes is… it’s not the actual conversation itself, that I don’t know what to say, but rather that you get such different reactions, you know? […] …they’re sensitive conversations. I mean, it’s not, it’s not just a question of whether they want hamburgers for dinner, it’s questions like, what do you want us to do if you get really sick? (Participant #8, district nurse)

Unexpected shifts in character of ACP conversations were described. Finding balance between humility and being straightforward in conversations was experienced as difficult. Professionals aspired to make residents and relatives feel safe during ACP conversations. In order to comfort relatives and help them prepare for their family member’s impending passing, professionals highlighted the need for relatives to be fully involved in the ACP process.

### Talking: a balancing act when juggling sensitive topics and various expectations

ACP conversations were experienced as difficult in touching on sensitive issues around death, and specifically focusing on residents dying in the foreseeable future. Professionals expressed that relatives were often not used to discussing existential issues or had anxiety around losing their family members which made conversations difficult.

Conditions which may hinder communication, e.g. dementia and hearing loss, were said to contribute to communication difficulties and influenced how ACP conversations in NHs unfolded. Even when residents could participate, it was reported that ACP conversations could be challenging since discussions could awaken thoughts and questions on other, unrelated topics that residents wanted to talk about.

It was understood that the way ACP questions were posed was of importance. Both open and more directed questions were utilised in ACP conversations, as were conscious strategies to guide conversations toward what was professionally judged to be most appropriate:

… it’s not like you just cut to the chase directly and ask about [do not resuscitate] and that kind of thing. You ask the relatives or the patient… ‘How do you feel about your current state of health? What’s important for you in the future?’ […] Sometimes you get explicit answers, other times not. And then you have to be a bit more direct and ask about, you know, hospital, intensive care, et cetera… But yeah, I usually try and steer the conversation to what I think is most reasonable. (Participant #7, physician)

Professionals emphasised the importance of relatives being well-informed of the purpose of ACP, implications of decisions made, and that decisions may be revised later. Professionals also highlighted the importance of having a realistic tone in conversations, conveying that physical bodies deteriorate, and life takes its course:

… relatives shouldn’t have to feel that they haven’t done all they can for their family member. That’s important. But there’s limits, you know. We can’t do much more. Life goes in one direction, the body gives up. […] But in the end it’s about making life as good as possible, at whatever stage that person’s in there and then. (Participant #3, nurse)

### Deciding: guiding decision-making

Professionals emphasised that ACP decisions were negotiated with residents and relatives. Residents with dementia were described as a particularly challenging group, with some professionals arguing that even residents with advanced dementia could participate in conversations given the right conditions, while others expressed that relatives had to take on a greater decision-making role when residents could not comprehend what was being discussed.

Professionals argued that physicians were ultimately responsible for decision-making, based on medical judgement of the most appropriate level of care. The general attitude expressed was that, while there were exceptions, most residents were severely ill and fared better cared for at the NH. It was thought that hospitalisation should generally be avoided, and symptoms alleviated in the NH. Questions of hospitalisation and resuscitation (or not) were said to take precedence over other broader issues such as quality of life:

In our organisation, that’s what… at least what I experience to be the two main focus points of advance care planning. […] It’s not like, what quality of life they want, if they want to see their… I’ve read the document we have here in the region. Pretty thoroughly anyway. But we don’t beat around the bush in these conversations talking about… ‘What gives them joy in life? How could we make their life better?’ …we don’t touch on that kind of thing here. Here it’s hospital or not, [do-not-resuscitate] or not. (Participant #6, nurse)

Professionals described how sometimes communication unknowingly faltered; that healthcare personnel and relatives believed they had reached an agreement but in fact had disparate understandings of decisions made. Professionals described frustration and panic among relatives when their family member later became critically ill, and no life-prolonging measures were taken:

That’s the risk, that you’re not on the same page but you think you are. And then, when the situation takes a bad turn, as it inevitably does, then they get so… can get frustrated that this isn’t what they agreed to. They haven’t understood the ramifications, that there’s their mum, lying there with a stroke, and suddenly they can’t communicate with her anymore, and they panic… (Participant #6, nurse)

There was a perceived risk that becoming too standardised in one’s approach could lead to unfounded decision-making with residents receiving generalised care plans, out of alignment with individual needs and wishes. This would be particularly disadvantageous for healthier residents. Professionals considered it good practice to revisit and reassess ACP decisions at regular time intervals since health status could quickly change.

### Sharing: documenting preferences and disseminating information

Professionals described challenges with documentation. Do-not-resuscitate was seen to be sometimes used incorrectly as a catch-all term for all DLTs, or for palliation, which could result in emergency actions being taken that were detrimental for residents. More attention to detail in documentation was called for:

There’s such a difference in quality from one advance care plan to another, how they’re written that is. It’s really important to be detailed. […] Those who aren’t used to working… methodically might just write [do-not-resuscitate], or [do not intubate]. And nothing else. That’s the care plan. […] It’s like, they haven’t really thought it through what [do-not-resuscitate] actually stands for. […] A lot of people read a lot more into it. Palliation, basically. But it really doesn’t mean that. (Participant #9, physician)

Professionals explained that physicians and nurses document in incompatible documentation systems, requiring the physician at the health centre to fax documentation to the NH nurse. The regional documentation system was said to be more accessible with regard to finding residents’ advance care plans. Nurses found accessing the same information in the municipal documentation system more time consuming.

Relaying documented decisions, in particular do-not-resuscitate and do-not-hospitalise decisions, to NH nursing auxiliary personnel was considered to be of utmost importance. Nursing auxiliaries were however not able to access the nurses’ documentation system, so dissemination was in some places carried out orally via weekly review meetings of all residents, or by having DLTs clearly marked and placed in residents’ medicine cabinets in their apartments:

For [do-not-resuscitate], we put together a document that we have back-to-back with patients’ medication list, visible to all staff that come near it. You can see which doctor made the decision, which nurse was present. And which relatives. So all the staff can see it. Other things in the care plan are in the patients’ journal. But it’s only the nurses that see that. (Participant #5, nurse)

## Discussion

The findings of this study showed engaging in meaningful ACP conversations to be a process of preparing, being, talking, deciding, and sharing. Shortcomings of the ACP implementation process are highlighted, resulting in incomplete adoption in some workplaces, such that routines were not fully integrated or maintenance of the practice was faltering. Hasty implementation sometimes led to considerable confusion around what ACP would entail, which, together with other challenges reported, e.g. discussing existential issues; executing ACP with residents with cognitive impairment; and meeting relatives’ demands and expectations, may be indicative of insufficient training. Batchelor et al. [25] found lack of knowledge to be disempowering for personnel in carrying out ACP conversations, while conversely, training was seen by Spacey et al. [26] to be fundamental in providing tools and knowledge to successfully work with ACP. However, solely focusing training on knowledge transfer is problematised by Gilissen et al. [27], as self-efficacy rather than knowledge, was shown to be associated with NH nurses engaging in ACP. Designing interventions to raise NH nurses’ ACP knowledge and self-efficacy may be difficult [28].

Professionals in this study argued that ACP implementation could be improved with facilitators who could oversee implementation, and coordinate training and support, similar to findings in other studies [29,30]. This also aligns with the integrated framework for Promoting Action on Research Implementation in Health Services, i-PARIHS, in which competent facilitation is seen as the active agent for successful implementation of innovation into working healthcare practice [31]. The framework describes competent facilitation as sensitive attunement to the particularities of different recipients (healthcare personnel, managers etc.) and their specific context, to tailor the intervention appropriately. Without named sensitivity to either recipients or context, significant barriers were experienced in this study, with confusion around what ACP would entail, and lack of knowledge and training. A facilitator, on the other hand, by working closely with recipients in context and understanding their needs, could have eased implementation by promoting such facilitating factors as were revealed in this study: clearer leadership, workable documentation systems and routines, improved collaboration, and greater engagement in ACP from all parties involved, both regional and municipal. A review by Gilissen et al. [32] found similar factors to be necessary for ACP implementation in NHs: supportive management, suitable documentation systems, sufficient training, time and resources for ACP, monitoring to evaluate performance, and good relationships between personnel, patients and relatives. These factors also point to the importance of viewing context with a wider lens, incorporating local, organisational and health systems levels. Such a system-level approach is essential to sound implementation [31]. Given the relative complexity of how residential elder care in Sweden is organised, with two separate healthcare providers, different documentation systems and contextual factors, as well as several professions that need to collaborate, an explicit implementation strategy in the manner of i-PARIHS would seem crucial for successful ACP implementation.

Findings showed that professionals were person-centred in their approach to ACP, yet simultaneously goal-oriented. The person-centred nature was articulated by professionals as employing sensitivity in ACP conversations and tailoring the content to whom they were talking to. This was particularly important when residents suffered from cognitive impairment. The goal-oriented nature was formulated by professionals as prioritising decision-making during physicians’ first home visit, and guiding conversations towards what was felt to be in residents’ best interests, based on professional medical judgement. Although somewhat of a juxtaposition, goal-orientation is not contrary to a person-centred approach. A Swedish report [33] states that the purpose of person-centred care is to reach agreement between patients, relatives and healthcare professionals about the goals of treatment, and to create an unambiguous care plan. Professional assessment of medical and nursing needs is indispensable to this end. This study brought to light, however, a more generalised medical viewpoint that, in most cases, residents were better cared for in the NH and that hospitalisation was to be avoided. While this is a viewpoint supported by research: death in hospitals has been associated with lower quality of end-of-life care compared to death in NHs [34], there is a potential risk of becoming too standardised, less person-centred, leading to some residents not receiving the most appropriate care for them, which professionals in this study also pointed to. Likewise, Johansson et al. [35] see the risk of conceptualising quality of care in terms of universal values based on assumptions and inference, rather than explicitly expressed individual end-of-life preferences. Conversational tools may be helpful in person-centring ACP conversations in NH settings [13,36].

Professionals saw it as imperative that nursing auxiliaries and nurses knew how to act in emergency situations with regard to each individual resident, thus making clear-cut decisions and available documentation as necessity, as also shown by others [37]. This may explain the focus on DLTs in this study’s findings rather than broader issues such as quality of life and death, views on dying alone or having relatives present, and spiritual beliefs. This finding is not unusual; Sussman et al. [38] found that 80% of tools intended to facilitate ACP conversations are exclusively medically focused. They contend however that implementation of ACP into NHs is less likely to be successful when psychosocial issues are omitted, reasoning that medical decisions alone cannot account for all eventualities, and that advance care plans based on patients’ values and beliefs provide clearer guidance in moment-to-moment decision making.

### Strengths and limitations

This study is the first to report on ACP implementation in NHs in Sweden from a multi-professional perspective. A limitation is the low number of participants; however, the narrow study aim and dense specificity of the sample supports information power for this exploratory analysis [39]. Low uptake of healthcare personnel in research is a common issue, largely due to lack of time and incentive [40]. Professionals’ reasons for not participating are unknown, but given they did not, means many experiences and views on ACP in the target region were not accounted for. However, including different professions with participants from different workplaces strengthened heterogeneity in data.

These findings are specific to the target region where the study was carried out and implementation of ACP routines is ongoing. The findings could be transferable to other workplaces in the region and could also be relevant in other regions looking to implement ACP in NHs. The peculiarities of the Swedish healthcare context, however, may limit transferability of the organisational level findings to healthcare systems in other countries, but any healthcare provider looking to implement ACP could use these findings to help overcome potential barriers and learn from facilitating factors.

## Conclusion

Successful implementation of ACP in NHs requires a carefully planned implementation strategy, ideally one that utilises a system-level approach with designated facilitators who can oversee and support the process. Our results also showed that ACP in NHs tend to be medically focused with less attention given to residents’ other psychosocial care-planning needs, thus limiting guidance for decision-making in a range of situations and prerequisites for person-centred care.

Widespread uptake of ACP in Sweden could be useful in the national effort to adopt more person-centred care in Swedish healthcare. In order to improve the delivery of ACP in Sweden, more research on the implementation processes in various settings, as well as patients’ and relatives’ experiences of ACP, is required.

## Supplementary Material

Supplemental Material
