# Supplementary material for: Implementing advance care planning in Swedish healthcare settings – a qualitative study of professionals’ experiences
Source: Scand J Prim Health Care. 2022 Dec 15;41(1):23–32. doi: 10.1080/02813432.2022.2155456 (PMC10088918; doi:10.1080/02813432.2022.2155456)
Supplement: Supplemental Material [file IPRI_A_2155456_SM5716.docx]

| 1. Would you like to start by telling us your thoughts on what advance care planning is and how it should be implemented? | |
| --- | --- |
| *Possible follow-up questions:* | - What is the purpose of advance care planning? - What can it lead to/result in? |
| 1. Please tell us about your experiences with advance care planning conversations? | |
| *Possible follow-up questions:* | - Can you explain what usually happens during advance care planning conversations? - Describe your experiences of holding advance care planning conversations with patients. - What kind of response have you received from patients and relatives? |
| 1. Can you think of any factors that make it easier to initiate and carry out advance care planning conversations? | |
| *Possible follow-up questions:* | - If you think of times advance care planning conversations went smoothly, what made them go so smoothly? - Is there anything in your organisation’s working routines or management that make holding advance care planning conversations easier? - Is there any kind of support you wish you had to help you in this work? |
| 1. Are there any factors that make it more difficult to initiate and carry out advance care planning conversations? | |
| *Possible follow-up questions:* | - What barriers or difficulties have you encountered when trying to initiate advance care planning conversations? - If you think of times you met resistance in advance care planning conversations or found them difficult, what was it that caused those difficulties? - Is there anything in your organisation’s working routines or management that makes holding advance care planning conversations more complicated? |
| 1. Is there anything else you would like to mention or discuss? | |
| *General questions:* | - Tell us more. - What do you mean? - Can you give an example? - What did you do? |
